# Implications of Dual Practice among Health Workers: A Systematic Review

**Published:** 2017-02

**Authors:** Javad MOGHRI, Arash RASHIDIAN, Mohammad ARAB, Ali AKBARI SARI

**Affiliations:** 1. Dept. of Management Sciences and Health Economics, School of Health, Management & Social Determinants of Health Research Center, Mashhad University of Medical Sciences, Mashhad, Iran; 2. Dept. of Health Management and Economics, School of Public Health, Tehran University of Medical Sciences, Tehran, Iran

**Keywords:** Dual practice, Health workers, Implications, Consequences

## Abstract

**Background:**

Mixed health care systems to work simultaneously on both public and private facilities, is common today. This phenomenon referred to as dual practice (DP), has potential implications for access, quality, cost and equity of health services. This paper aimed to review systematically studies that assess the implications of DP among health workers.

**Methods:**

MEDLINE, EMBASE, and The Cochrane library were searched for obtaining published literature between Feb 1990 and May 2014. Google and Google Scholars, organizational websites, and reference lists of relevant papers searched to get grey literature. Only studies concentrated on consequences and impacts of DP among health professionals and conducted using “randomized controlled trials”, “non-randomized controlled trials”, “controlled before and after studies”, or “interrupted time series” were eligible for inclusion.

**Results:**

From 3242 records, we focused on 19 studies, which aimed to assess effects and impacts of dual practice. After that, the current understanding of DP positive and negative implications was categorized and discussed based on two perspectives.

**Conclusion:**

There has been a propensity to over-reliance on theoretical methods in predicting the implications of this phenomenon. Almost all of the mentioned implications are based on theoretical predictions undermined in the broader literature. Furthermore, assessing the current literature showed positive and negative impacts of DP on different parts of the health system and various dimensions of service delivery. These implications are contexted specific and may vary from system to system.

## Introduction

Today, the private health sector plays a significant role in service delivery besides the public sector. The contribution of private sector in health care is ranging from providing services for 14% of the population in Thailand to 70% in Zimbabwe (1). Unregulated growth of the private sector, limited human resources, low salaries and poor working conditions in public sector have made an attractive opportunity for public sector health workers to seek their unmet needs and expectations in the other sector (1, 2). Now, it is common for health workers in general, and specifically for physicians in many countries with mixed health care systems to work simultaneously at both public and private facilities (3–7). This phenomenon referred to DP, is common in many developing and developed systems such as UK, Australia, China, Peru, Zambia, etc. (2, 8–10), and has gained many attentions from the early of the new millennium.

DP has both negative and positive implications in developing and high-income countries, debates on the negative effects by far exceed the positive (4, 11). Allowing DP can serve to supplement low public salaries and help governments to recruit and retain health workers in public facilities even in rural and remote areas without extra-budgetary burden, which could escalate access to services in these locations (1, 12, 13). Working in the private sector enhances technical knowledge and skills of government providers, boost public services quality (12). Dual practitioners have incentives to do their best and give quality services in their public job to get a good reputation and advertise for their private practices (12). Dual practitioners have incentives for “price discrimination” by shifting rich patients from public to private sectors and therefore reducing the public waiting lists and increasing access for both the poor and non-poor (12). On the other hand, health workers engaging in both public and private sectors may shirk their public sector duties, and reduce the quantity and quality of their services in this sector -due to their conflict of interest- and favor long waiting times in public healthcare facilities to boost demand for the private counterpart (1, 4, 5, 14, 15).

Competition for time has been mentioned as the other problem associated with DP, where a considerable proportion of dual workers are believed to reduce their work hours in the public sector to limit access by public patients (1, 9, 16). Besides, competition for time, illegal and uncountable hidden outflow of resources such as means of transportation, drugs, personnel, and sundries from public to private practice is the another problem attributed to DP (1). Dual practitioners are also suspected to cream-skim rich or low complicated patients from public sector and shift them to their private working places or prescribe unnecessary services for their own profit (5, 14, 17, 18).

The lack of social consensus on this subject is reflected by various governments’ responses toward DP (4). These diverse interventions and policies include banning DP on one side, to allowing it without any restrictions on the other side of the spectrum (19). Moreover, there has not been any scientifically reliable published document about the actual effects of these managing strategies (7).

The second main research priority into human resources for health in low and middle-income countries is “What is the impact of DP and multiple employment?” (20). Policymakers and health officials all over the world need meticulous evidence about the factual consequences and impacts of DP to design appropriate interventions and policies for management of this phenomenon.

Despite the importance of the subject, there is no evidence that systematically review the consequences and impacts of DP. This article aimed to review the related studies in this field and assess the implications of this phenomenon systematically.

## Methods

### Study design

Systematic review methodology was used to assess the consequences and impacts of DP among health workers.

### Search strategy

The following electronic databases were searched: MEDLINE, EMBASE, and The Cochrane Library. Due to the fresh nature of the topic in health sector, our searches were limited to studies published between Feb 1990 and May 2014. The search strategy was tested for the sensitivity of finding known relevant studies and altered accordingly. In order to capture all relevant papers, we decided not to use design filters or combine words related to special designs in our electronic search. The general format for the final search strategy was “DP related terms”, “terms which are relevant to major health professions”, and “terms related to the consequences and impacts of DP”. This search strategy was devised for use in Medline (accessed via PubMed) and then it was translated into the other databases using the appropriate controlled vocabulary, as applicable.

Google, the Google Scholars search engines, conducted targeted search in organizational web-sites including World Bank, WHO, Global Human Resources for Health resource center, the Human Resources for Health Online journal were searched to ensure that potentially relevant studies were not missed. In addition, reference lists of identified, relevant papers were also checked for obtaining further related documents.

### Inclusion criteria

Only studies concentrated on consequences and impacts of DP among health professionals and had been conducted using randomized controlled trials (RCTs), non-randomized controlled trials (nRCTs), controlled before and after (CBA), or interrupted time series (ITS) methodology were eligible for inclusion and further consideration. Moreover, for practical reasons such as time and financial limitations, studies written in English or Persian were considered.

### Selection process, data extraction, and analysis

Our electronic searches were entered into the EndNote X4 software. At first, one author (JM) assessed the titles and then abstracts to exclude clearly unrelated ones. After that, full copies of the remaining papers were ordered for assessing eligibility for inclusion. In the next step, two authors (JM, and AAS) evaluated full texts independently. Any disagreements between reviewers were resolved through discussion and consensus. Finally, JM extracted data from the included studies, and AAS assessed the completeness and accuracy of the data extraction.

## Results

**Fig. 1:**
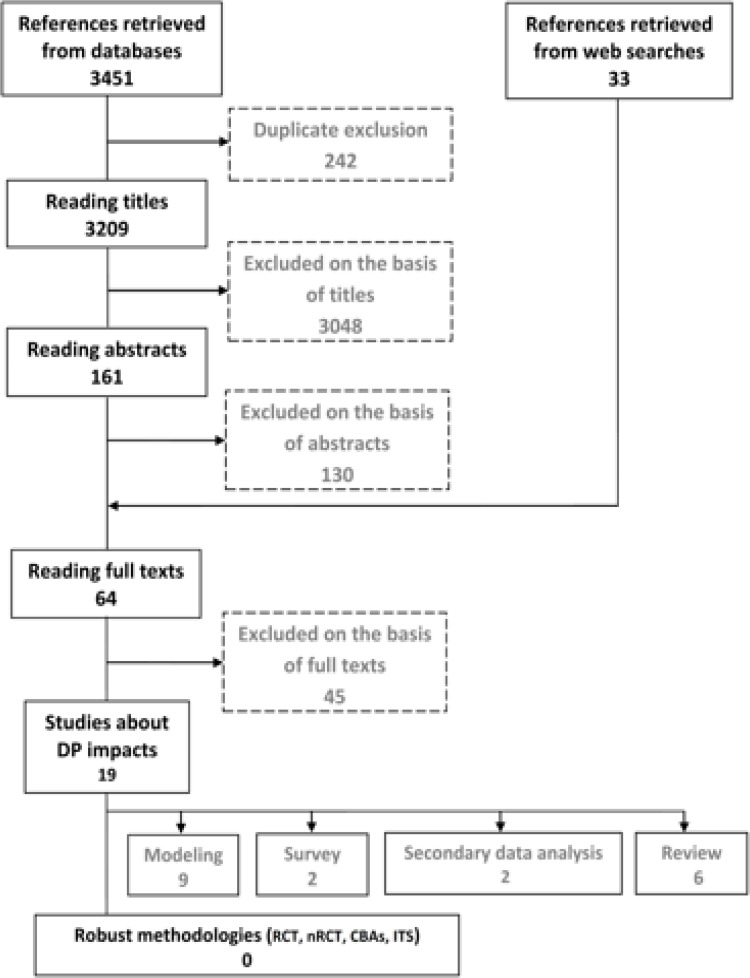
Paper selection flowchart

Totally, 3209 titles and 161 abstracts from database searches were assessed. After adding 33 references from web searches and backward citations, we examined 64 full-text reports and papers. At the end, 19 of them were recognized as relevant and acceptable in terms of appropriateness and quality, from which no one was eligible for inclusion according to the criteria of this review (Fig. 1). These studies are marked as “relevant studies” and comprised of nine papers using modeling technique, two surveys, two secondary data analysis, and six reviews (Table 1). Our searches could not yield any studies which use vigorous methodologies and research designs (RCTs, nRCTs, ITS, and CBA) for assessing effects, consequences, and impacts of DP among health workers.

**Table 1:** List of the relevant studies

**Author (yr) (Ref. No.)**	**Method**	**Context**	**Main Results**
Barros & Oliviera. (2005) (21)	Modelling	HICs*	1. “Physicians do not necessarily end up treating the mildest cases from the waiting list” (because only more severe patients in the waiting list are willing to use private services and pay for it).
Biglaiser & Ma (2007) (15)		HICs	1. “Allowing moonlighting always enhances aggregate consumer welfare, but equilibrium public-care quality may increase or decrease”.
			2. “Unregulated moonlighting may reduce consumer welfare as a result of adverse behavioral reactions, as moonlighters shirking more and dedicated doctors abandoning their sincere behavior”.
Bir & Eggleston (2003) (18)		LMICs*	1. “Governments can meet the participation constraint of physicians without paying salaries to commensurate to physicians’ abilities because physicians also value the “non-salary benefit” of the opportunity to earn significant private practice revenues”.
			2. “If dual-practitioner differentially refers higher income patients to private practice, public funding becomes more effectively targeted on the poor. However, physician incentives to concentrate inducement on those most responsive to inducement -often the poor and uneducated–may act counter to such a social objective”.
Brekke & SØrgad (2006) (22)		HICs	1. “Allowing physician DP ‘crowds out’ public provision and results in lower overall healthcare provision”.
Gonzalez (2004)(23)		Both HICs and LMICs	1. “Physician will have incentives to over-provide medical services when he uses his public activity as a way of increasing his prestige as a private doctor”.
			2. “Physicians’ DP can be either welfare improving or reducing, depending on the treatment policy that the health authority wants to implement (If the priority is to contain costs, then the doctor’s dual activity is negative. If the priority is to minimize patients’ health losses, his DP affords the objective at a lower cost)”.
Gonzalez (2005) (24)		Both HICs and LMICs	1. “When physicians are dual providers, the problem of cream skimming arises”.
Iversen (1997) (25)		HICs	1. “Without rationing of waiting-list admissions, a private sector is shown to result in a longer waiting time if the demand for a public treatment is sufficiently elastic with respect to the waiting time. When waiting list admissions are rationed, the waiting time is shown to increase if the public sector consultants are permitted to work in the private sector in their spare time”.
Jiwei (2010) (26)		LMICs	1. “Allowing DP can improve welfare through escalating efficiency in two ways: first, resource allocation within the hospital is more efficient; second, allowing DP can save salary expenditure for the public hospital”.
			2. “After allowing dual practice, rich patients with mild cases are more likely to be induced to private clinics from the hospital. Low-income patients, or patients with serious conditions, are more likely to be treated in the hospital. Therefore, physician DP can also be interpreted as an alternative instrument for sorting in terms of both illness severity and switching costs”.
Morga & Xavier (2001) (27)		HICs	1. “The presence of ‘selfish’ behavior among dual practitioners was found to lead to: a) a decrease in the optimal number of patients treated as NHS elective surgery cases; b) An increase the waiting time NHS elective care patient’s face; c) an increase in health care costs”.
Hanvoravongchai et al. (2000) (28)	Survey	LMICs	1. “Private practice could lead to deterioration of public confidence in obstetric services in public hospitals, and consequently it encourages a move to private practice”.
			2. “Private practice, whereby physicians feel obliged to provide personal delivery services when triggered by leisure and time conflict, leads to higher and possibly unnecessary cesarean procedures”.
Socha & Bech (2011) (11)		HICs	1. “Results do not reveal any patterned relationship between DP and public hospital work hours, participation in voluntary tasks or activities that might conflict with the private-practice hours, or preferences for part-time employment”.
			2. “Results also do not support the general presumption that the physicians who work exclusively in public hospitals are more altruistic and hence, voluntarily provide more work inputs than dual-practitioners”.
Bloor et al. (2004) (29)	Secondary data analysis	HICs	1. “Consultant surgeons with a ‘maximum part-time’ contract had significantly higher activity rates than those with a full-time contract”.
Johannessen & Hagen (2014) (30)		HICs	1. “The total working hours in public hospitals were similar for both those who did and did not engage in DP (in different tasks such as the planned working hours, on call duties, and overtime work); however, DP reduced public working hours in some specialties with significantly higher private incomes”.
Eggleston & Bir (2006) (8)	Review	Both HICs and LMICs	1. “All theories to date suggest that the impact of DP on public service quality is ambiguous”.
			2. “The social trade-off between the benefits and costs of DP hinge on the quality of a country’s contracting institutions”.
			3. “Allowing DP may improve social welfare and the quality of public services, under specific circumstances”.
			4. “The evidence does not support the perception that ‘full-timers’ embody greater commitment and contribution to public sector provision”.
Ferrinho et al. (2004) (1)		LMICs	1. “Negative impacts are predatory behavior (self-gain is preferred to the interests of others), conflict of interest (lower the quality in the public sector to advertise for the private sector), brain drain (to other countries, private sector, or urban areas), Competition for time and limits to access, Outflow of resources and corruption (illegal use of public resources for private patients)”;
			2. “Positive impacts are the ability to generate additional income for health workers, and higher professional satisfaction”.
Hippgrave et al. (2014) (31)		LMICs	1. “Lack of information from studies at the country level, tendency of reviews to rely on secondary data, and rapidly changing environment made it very difficult to write with confidence about the impacts of DP among nations in South and East Asia”.
			2. “Positive and negative impacts of DP have been mentioned in different studies in the region. Positive effects were improving access through parallel supply chains and expand services in terms of hours of availability and provision of health care in rural areas, improving the satisfaction of health workers (increasing income, prestige, etc), improving equity of access and system efficiency through sorting of patients, and etc. Negative aspects were decreasing access and equity through absenteeism in the public sector and the tendency of workers to migrate to urban areas (more DP opportunities compared with rural areas), prioritizing better off patients and neglecting the other group, cream skimming, and etc”.
			3. “ In market economies with limited public sector capacity, well-regulated DP probably improves health service access and possibly its efficiency”.
Jan et al. (2005) (14)		LMICs	1. “DP can be a possible system solution to issues such as limited public sector resources, low regulatory capacity, and the interplay between market forces and human resources”.
Prado & Gonzalez (2011) (9)		Both HICs and LMICs	1. “While dual providers may be tempted to skimp on time and effort in their main job, to induce demand for their private services, or to misuse public resources, the legalization of DP may also contribute to recruit and retain physicians with less strain on the budget and improve access to health services, especially in developing countries”.
			2. “The implications of DP that are important to one country are not necessarily important to others”.
Socha & Bech (2011) (5)		Both HICs and LMICs	1. “Theoretical analyses indicate both positive and negative effects of dual practice”.
			2. “Some of the effects depend on assumptions that are undermined in the broader literature (e.g. the intention to maximize income)”.

* HICs: High-income countries. LMICs: Low-and-Middle-income countries.

### Quality assessment and risk of bias

We had evaluated methodological quality of included evidence using EPOC (Prepared by Effective Practice and Organization of Care (EPOC) group, and has been used in many reviews for checking special design studies against bias) risk of bias checklists for RCTs, nRCTs, ITS, and CBA studies, but no documents were qualified for inclusion in this study.

However, the quality of 19 relevant studies was evaluated using a self-developed simple assessment tool. This tool contained some questions about the methods of conducting studies, study setting and sample, data collection process, the process of data analysis, and other questions. Almost all of these documents were subjected to potential biases. For example, in all of the studies, which used modeling technique for providing a theoretic prediction of the impacts of DP on different aspects of health, researchers supposed assumptions questioned in the broader literature, or belong to special markets and environments (5). In addition, with neglecting the personal characteristics differences of single and dual practitioners in the quantitative studies, there is a potential risk of bias in these researches. However, due to the non-existence of any rigorous evidence, we presented a summary of key findings of these studies in this section (Table 1), and discussed them further in the next part of the paper to portray a picture of evidence and their findings in this area. Besides, these 19 studies have exposed to some biases, all of them are the most quality evidence on impacts of DP that are available to now.

### Consequences and impacts off dual practice

**Fig. 2:**
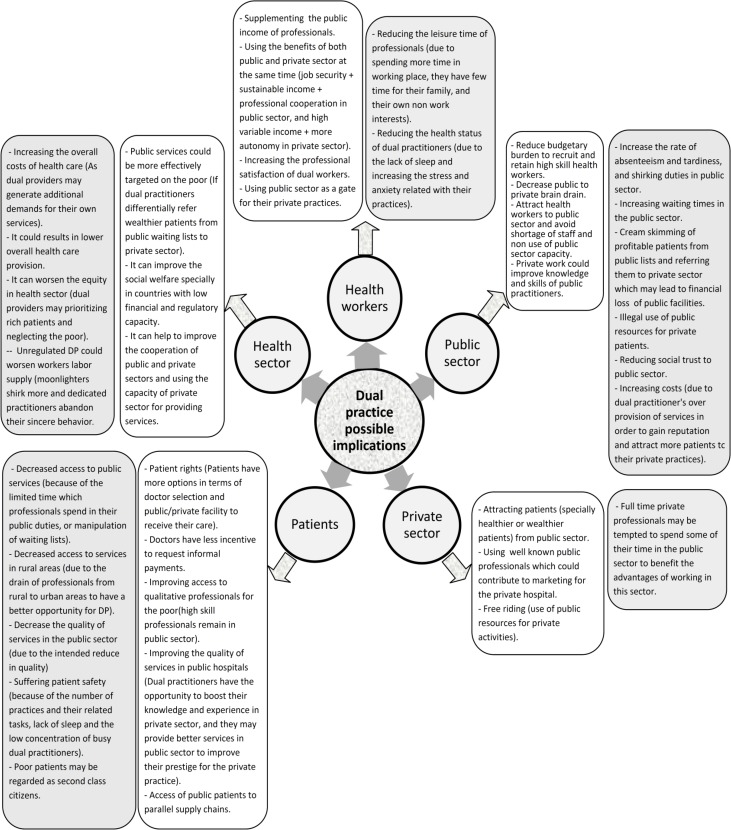
Dual practice possible implications on patients, health workers, public sector, private sector, and overall health sector (The meaning of background color of rounded rectangles: white (Possible positive impacts) and gray (Possible negative impacts))

Our electronic searches found 3242 hits (3209 titles from electronic database searches plus 33 references from web searches and backward citations), of which none were eligible for inclusion in this review after screening. A list of 19 main studies in this field and their important findings are provided in Table 1. Moreover, the possible implications of DP on patients, health workers, public sector, private sector, and overall health sector are shown schematically in Fig. 2.

## Discussion

We conducted a systematic search and assessed different documents in the field of DP implications and impacts. Although, the subject has been identified as one of the most important research priorities especially in low and middle-income countries (20), our searches could not found any robust evidence assessing the real consequences and impacts of this phenomenon. However, 19 relevant (not included) studies were considered to describe and discuss the current understanding of this subject and draw a picture of evidence in this field.

The scope of possible positive and negative impacts of DP on patients, health workers, public sector, private sector and overall health sector are shown in Fig. 2. Furthermore, possible impacts of DP on four main aspects of health services delivery (access, quality, cost, and equity) are categorized and mentioned in the text.

### Access to services

There is not any robust evidence about the impacts of DP on patients’ access to services and the existing literature on this subject is inconclusive. This phenomenon could decrease access to services because of the absenteeism and dual providers skimping on time and efforts in the public sector (1, 9, 22, 31–35), or the increase in public sector waiting times (9, 25, 27, 32, 36, 37), or the drain of physicians from rural to urban areas seeking for better DP opportunities, which might suffer access in the rural areas (1, 31, 38). On the contrary, there are studies about the positive effects of DP on access to health services. For example, allowing DP could improve access to high quality professionals in the public sector (9, 14, 32, 39), or improving access through provision of services outside formal public working time in private sector or in rural areas (9, 17, 31, 32, 40, 41), or taking advantage of “parallel supply chains” when the necessary drugs and other requirements are absent in the public sector (31).

These results have come from studies in various countries and health systems, and they might be quite different in other contexts. For example, almost all of the studies that refer to the effects of DP on public sector waiting times represent national health systems with a powerful and quality public sector that may not applicable in other health systems. Therefore, most of the studies, which concluded the negative impact of DP on rural to urban drain of physicians, or on absenteeism and skimping of time and efforts in the public sector, represent low and middle-income countries (LMICs) with poor public sector environment and limited monitoring and supervision capacities. These factors may have substantial contribution to the negative implications of the phenomenon in these nations, while in well-developed systems these kinds of impacts are not usual. On the other hand, almost all of the mentioned positive consequences of DP are from LMICs studies. In fact, in these countries, dual job holding of health professionals is viewed as a possible system solution to the poor public sector condition, limited infrastructural and regulatory capacity, etc. (14).

### Quality of services

Decreasing the quality of public services is one of the most important reasons which DP critics refers to, in order to justify the related bans and restrictions (1, 14, 15, 19, 42). However, only this opinion is not based on rigorous evidence, but there are completely opposite results. Dual practitioners concentrate their efforts on private practice and do not pay enough attention to their public duties, which could lead to a reduction in public service quality (1, 15, 34). Dual practitioners may have conflict of interest and intentionally decrease their service quality in public sector in order to attract more patients for their private work, leading to a gradual reduction of social confidence in the former sector (1, 28, 38, 43). Dual practitioners have incentives to do their best in diagnosing patients in the public sector to gain prestige for their private practice (23). Allowing DP helps public sector to recruit and retain quality practitioners and use their good knowledge and skills in this sector (8, 9, 18, 44, 45). DP provides a unique opportunity for practitioners to “learn from a broader range of experience and colleagues” and enhance their knowledge and skills (9, 12).

Again, these studies present results from various settings. While in the first case of negative impacts, there are studies from both developing and developed studies, all of the mentioned studies in the case of conflict of interest are from LMICs. In underdeveloped systems, poor management and lack of monitoring and punitive mechanisms, creates a good condition for opportunistic dual practitioners to delegitimize the public sector in favor of their private activities, but it is not common in well-developed systems. For potential positive implications of DP, there are studies from both settings. By allowing DP as a non-pecuniary benefit, public hospital managers can attract and maintain proficient practitioners in their facilities and these professionals have the opportunity to promote their expertise, and these benefits are not limited to a specific system.

### Efficiency and cost of services

There are arguments about the impacts of DP on efficiency and cost of services in the literature. Again, lacking meticulous studies and scientifically reliable evidence on this subject, there is controversy and disagreement about the net effects of DP on efficiency and service costs. Dual practitioners cream skim more profitable patients from public sector and shift them to private sector, which results in a financial loss to the former sector (24, 31). Induces additional demands for their own services, leading to an increase in total health costs (1, 9, 28, 46). Dual practitioners over provide services in the public sector to gain prestige for their private practice (23), or DP could lead to corruption and out follow of resources from public to private sector (1, 9). Allowing DP could minimize public sector costs to employ and maintain professional health staff and avoiding human resources shortages and nonuse of public sector equipment and other service capacities(9, 14, 18, 26), or it could improve the efficiency in allocation of resources in hospitals (26).

Some negative effects of DP on the efficiency and cost of services are important in every setting regardless of the level of income and development, while others are significant only in LMICs. For example, over provision of services, or induced demands could be seen almost in every country less or more. Corruption and outflow of resources from public to private sector are more prevalent in developing and especially underdeveloped settings. This is because of nonexistence or poorly implemented management and control mechanisms in these countries, paving the way for opportunistic dual providers to use public resources for their own profit illegally.

### Equity of services

DP impacts on equity of services are one of the most important research questions unanswered so far. Again, there are arguments both for and against this subject, but there is no good quality information in this field. Dual practitioners may prefer well off patients and neglect the poor in public sector and regarded them as second-class citizens (28, 31, 47–49), or allowing DP could lead to absenteeism or migration of practitioners from rural to urban areas seeking for better DP opportunities, which might suffer equity of access and unequal distribution of practitioners in rural and underserved areas (1, 31, 38, 50, 51). Shifting rich patients to private sector by dual practitioners could lessen pressure on public sector and help it to more effectively targeted services to the poor (9, 18, 31), or allowing DP could provide incentives for proficient practitioners to stay in public sector and provide quality services for the poor (9, 18).

Almost all of the above studies have been done in LMICs context. Although, some of these implications might be seen in well-developed and high-income countries, they are of paramount importance in low and middle-income countries. There are a variety of arguments both for and against the effects and impacts of dual practice, made it unclear and complicated to reach an agreement about the net implications of the phenomenon. However, implications of DP could be different and even opposite in various settings. In some countries, DP is regarded as a problem, while it could be a potential system solution in other countries with limited infrastructure, financial and administrative capacity (14). In addition, regulation of DP and the quality of enforcement could be a major determinant of the effects and impacts of the phenomenon in each country. Furthermore, there are differences between various specialties about dual practice. In some specific fields of medicine, there are not major performance differences among single and dual practitioners, while in other specialties there are (9, 30). The extent of private practice opportunities, the level of income from such activities, and the gap between public and private earnings plays an important role in decision of professionals to divide labor between these two sectors and the quality of performing related tasks.

The important finding of this study was the nonexistence of robust and reliable evidence about the implications of DP among health workers, despite the importance of and need for it in order to adopt appropriate policies and interventions. Although, conducting rigorous studies with special designs on the issue of DP is difficult and rather practically challenging, but using national policies on DP as a natural intervention for experimental and quasi-experimental studies could be more feasible and produce more reliable evidence compared with the totally theoretic methods. Lacking quality evidence, there has been a propensity to over-reliance on theoretical and computerized methods like “modeling” in predicting the effects and impacts of this phenomenon. Almost all of the mentioned implications are based on anecdotal evidence and theoretical predictions undermined in the broader literature (5). Furthermore, there is not any significant difference between the performance of dual practitioners and full timers (8, 11,51), or dual practitioners even have better performance in the public sector than their full-time counterparts (29). Although, the methodologies used in these studies like other researchers in this field makes them vulnerable to important biases, they again mark the complex and conflicting nature of DP impacts.

Although meticulous methods were followed in this review, the search should not be considered all-inclusive and several limitations to this study need to be acknowledged. Firstly, we considered only studies reported in English or Persian because of practical reasons such as time and financial limitations. Secondly, only Medline, Embase, and The Cochrane Library databases were searched for the same reasons. Although we might miss some possibly exciting studies including non-English/Persian papers and reports, and studies indexed in other databases or published in local or non-indexed journals, authors tried to capture potential relevant studies through searching in the reference lists of important papers and googling in the search engines. Furthermore, adopting the strict inclusion criteria in our review led to finding no appropriate publications in this field, there is a major lack of reliable evidence on this important study issue.

## Conclusion

This is the first study that systematically reviews the evidence on implications of DP among health workers. Despite the paramount importance of the subject, there is not any robust evidence in this field. DP is a versatile phenomenon and has dissimilar implications in different contexts and settings and there is not a single recipe for it. Understanding the nature, prevalence, reasons, and the implications of DP is the first step toward designing appropriate policies and interventions for management of this phenomenon. Accurate diagnosis and estimation of DP potential negative implications in each context and settings could help policy makers to prescribe proper remedies for the related problems. Meticulous empirical studies using high quality experimental or quasi-experimental designs should be devised to detect the real effects and impacts of dual practice. National policies could be served as natural interventions and help researchers in designing such studies. In addition, reliable comparative studies could be designed to better portray the differences of DP implications between health systems, settings, and specialties.

## Ethical considerations

Ethical issues (Including plagiarism, informed consent, misconduct, data fabrication and/or falsification, double publication and/or submission, redundancy, etc.) have been completely observed by the authors.
